# Supplementary material for: Neutron reflectometry and NMR spectroscopy of full-length Bcl-2 protein reveal its membrane localization and conformation
Source: Commun Biol. 2021 Apr 27;4:507. doi: 10.1038/s42003-021-02032-1 (PMC8079415; doi:10.1038/s42003-021-02032-1)
Supplement: Supplementary file 1 — Supplementary Information [file 42003_2021_2032_MOESM1_ESM.pdf]

Neutron reflectometry and NMR spectroscopy of full-length Bcl-2 protein reveal its membrane localization and conformation

Ameeq Ul Mushtaq[1,a] Jörgen Ådén[1,a] Luke A. Clifton[2], Hanna Wacklin-Knecht[3,4], Mario Campana[2], Artur P. G. Dingeldein[1], Cecilia Persson[5], Tobias Sparrman[1] and Gerhard Gröbner[1,*]

Supplementary Information

Supplementary Figure 1: Organization of lipid membranes in the presence of full-length human Bcl-2 protein at a 70:1 lipid-to-protein ratio (d-Bcl-2:h-DMPC contrast).

Supplementary Figure 2: Organization of lipid membranes in the presence of full-length human Bcl-2 protein at a 70:1 lipid-to-protein ratio (h-Bcl-2:d-DMPC contrast).

Supplementary Figure 3: Titration of Bax-BH3 peptide against Bcl-2 protein.

Supplementary Figure 4: Titration of Nur77 peptide against Bcl-2 protein.

Supplementary Figure 5: Partial assignment of Bcl-2 residues.

Supplementary Figure 6: ^{1}H-^{15}N TROSY-HSQC spectra of Bcl-2 ΔTM protein (without the transmembrane C-terminal helix).

Supplementary Figure 7: Comparison of ^{1}H-^{15}N TROSY-HSQC spectra and tryptophan NMR signals of Bcl-2 and Bcl-2 ΔTM proteins in DPC micelles.

Supplementary Figure 8: Comparison of ^{1}H-^{15}N TROSY-HSQC spectra and tryptophan NMR signals of Bcl-2 and Bcl-2 ΔTM proteins in the presence of DPC below CMC (1.5 mM).

Supplementary Figure 9: Titration of Bax-BH3 peptide against the Bcl-2 ΔTM protein variant.

Supplementary Figure 10: Titration of Bax-BH3 peptide against the Bcl-2 ΔN(1-82) protein variant.

Supplementary Figure 11: Titration of Bim-BH3 peptide against the Bcl-2 ΔN(1-82) protein variant.

Supplementary Table I: Known and calculated Scattering Length Density (SLD) values for components of the interfacial samples used in the NR studies.

Su**pplementary References**

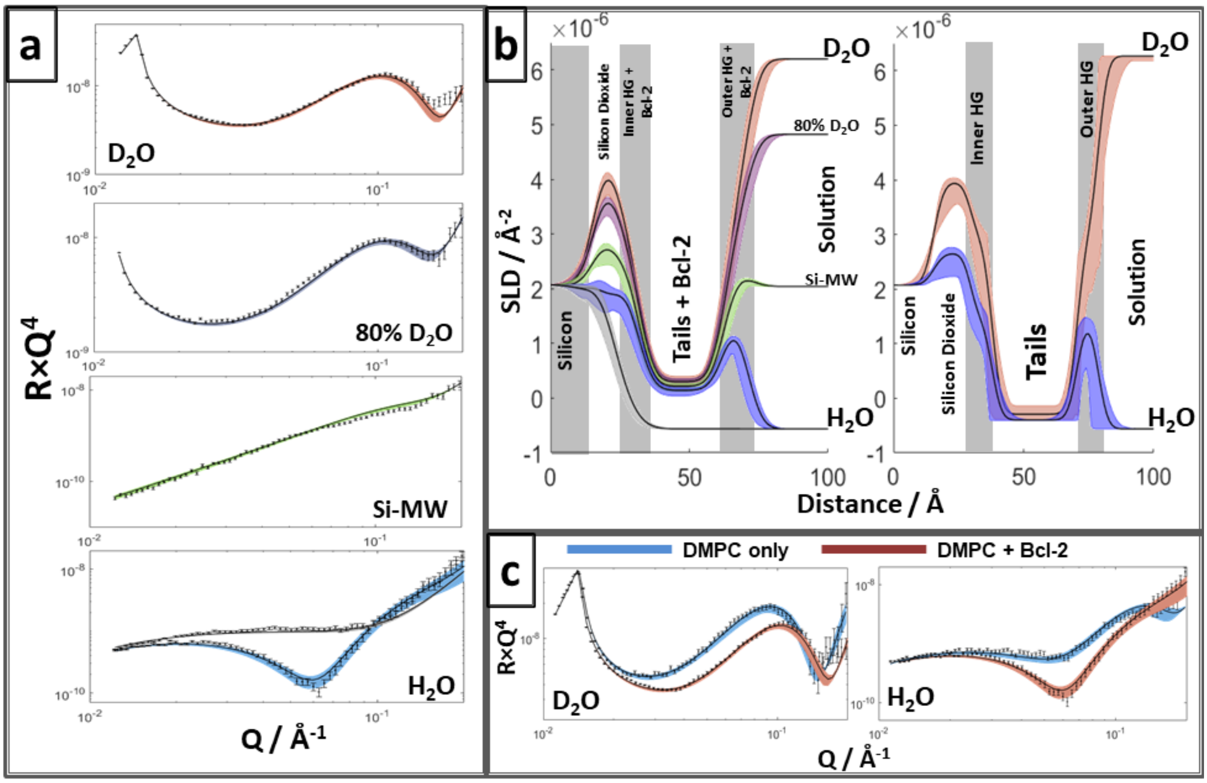

Supplementary Figure 1. Organization of lipid membranes in the presence of full-length human Bcl-2 protein at a 70:1 lipid-to-protein ratio (d-Bcl-2:h-DMPC contrast). a Experimentally obtained neutron reflectivity profiles (error bars) and model data fits (lines) obtained from a d-Bcl-2:h-DMPC supported lipid bilayer at the silicon/water interface analyzed under multiple solution isotopic contrast conditions. Data and fits for the silicon substrate alone in H_2O (grey line) shown for comparison. **b** Scattering length density profiles obtained from these fits (left) with profiles (shown for comparison) obtained for a DMPC bilayer alone (right). **c** Comparison of experimental profiles for DMPC bilayers with and without Bcl-2 derived from the highest lipid and protein contrast data, highlighting effects of Bcl-2 in the bilayer on the reflectivity profiles. Line widths in both the fits and SLD profiles represent the 95% confidence intervals obtained by MCMC sampling of the model-to-data fits.

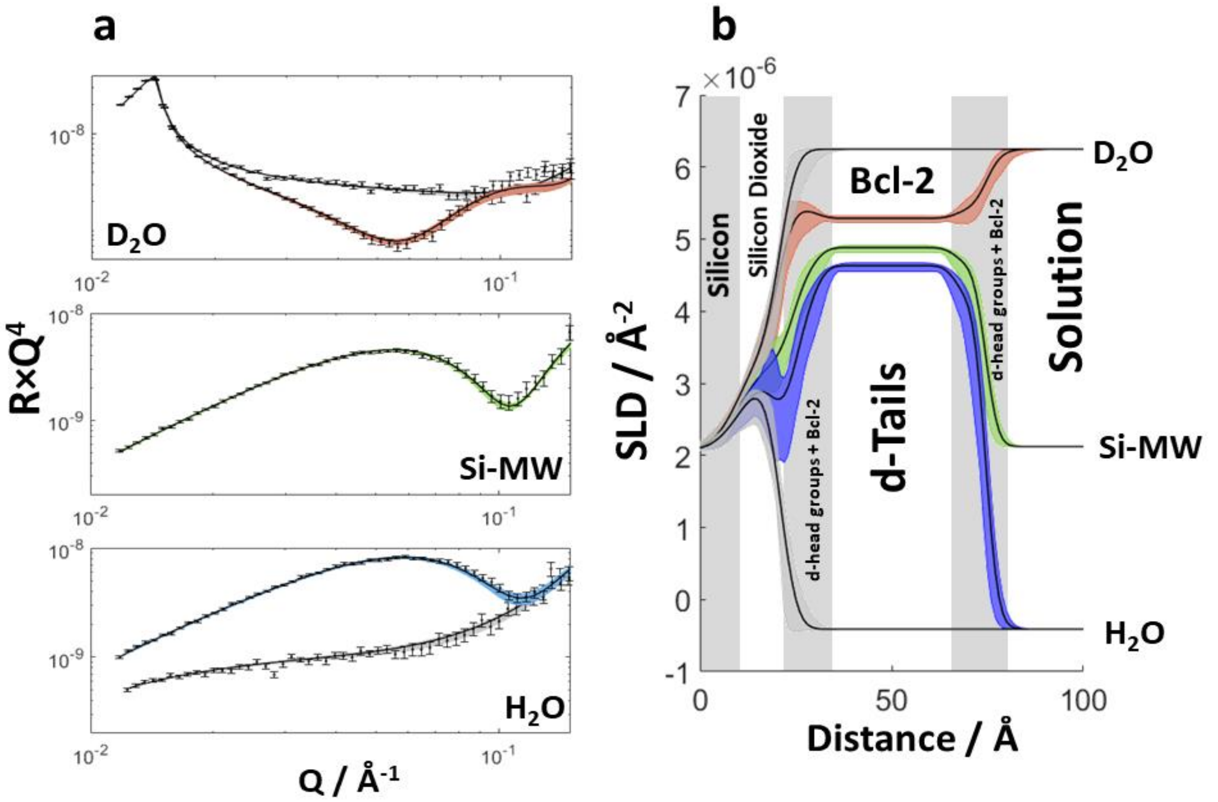

Supplementary Figure 2. Organization of lipid membranes in the presence of full-length human Bcl-2 protein at a 70:1 lipid-to-protein ratio (h-Bcl-2:d-DMPC contrast). a Experimental neutron reflectivity profiles (error bars) and model data fits (lines) obtained from a h-Bcl-2:d-DMPC (head and tail labelled) supported lipid bilayer at the silicon/water interface analyzed under multiple solution isotopic contrast conditions. **b** Scattering length density (SLD) profiles obtained from these fits, with labels used to highlight features of the protein and lipid components of the complex structure. Line widths in both the fits and SLD profiles represent 95% confidence intervals obtained by MCMC sampling of the model-to-data fits.

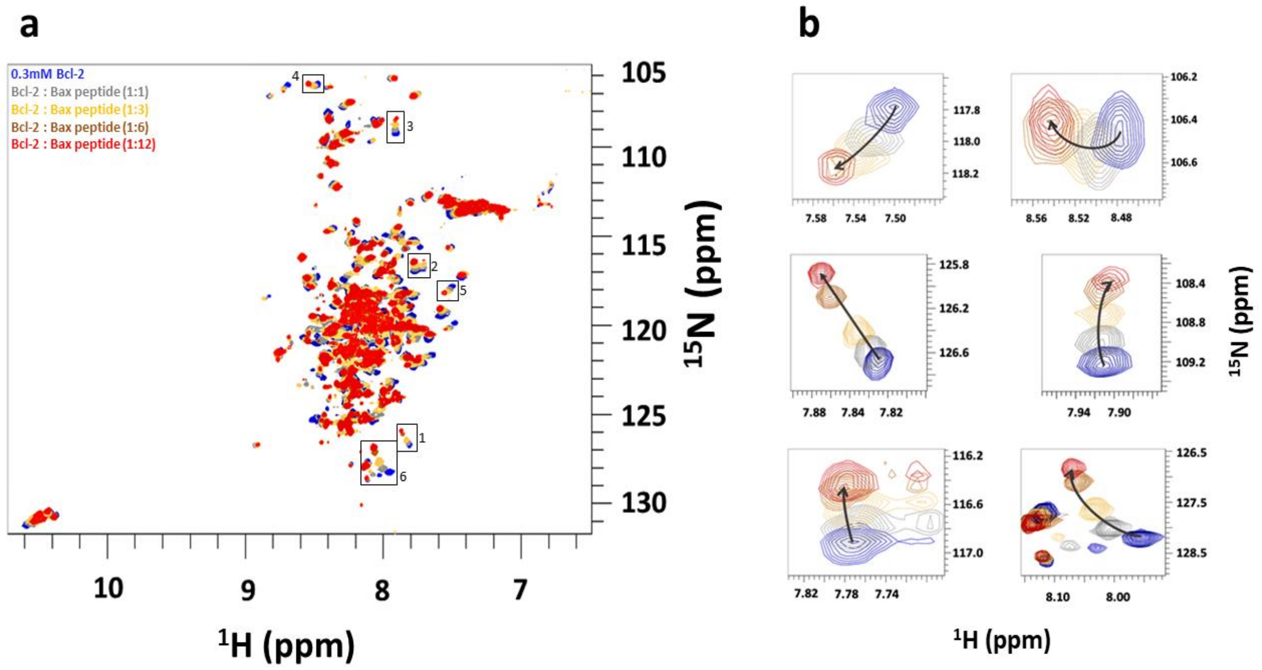

Supplementary Figure 3. **Titration of Bax-BH3 peptide against Bcl-2 protein. a** Overlay of ^{1}H-^{15}N-TROSY-HSQC spectra showing chemical shift perturbations (CSP) observed in ^{13}C/^{15}N/^{2}H-labeled Bcl-2 upon titration with the Bax-BH3 peptide. The spectrum of 0.3 mM Bcl-2 in 5 mM DPC micelles, 20 mM NaPi, 20 mM NaCl, 2 mM TCEP at pH 6.0 is shown in blue. Spectra of Bcl-2 with Bax-BH3 peptide added at 1:1, 1:3, 1:6 and 1:12 molar ratio are shown in grey, chrome-yellow, brown and red, respectively. **b** Zoomed boxes show the significantly perturbed peaks of the full Bcl-2 spectrum upon addition of the Bax-BH3 peptide. For the residues yielding signals shown in the top left of Figure B, top left, a K_D of 143±28 μM was determined. Spectra were acquired at 310 K and at a ^{1}H frequency of 850 MHz.

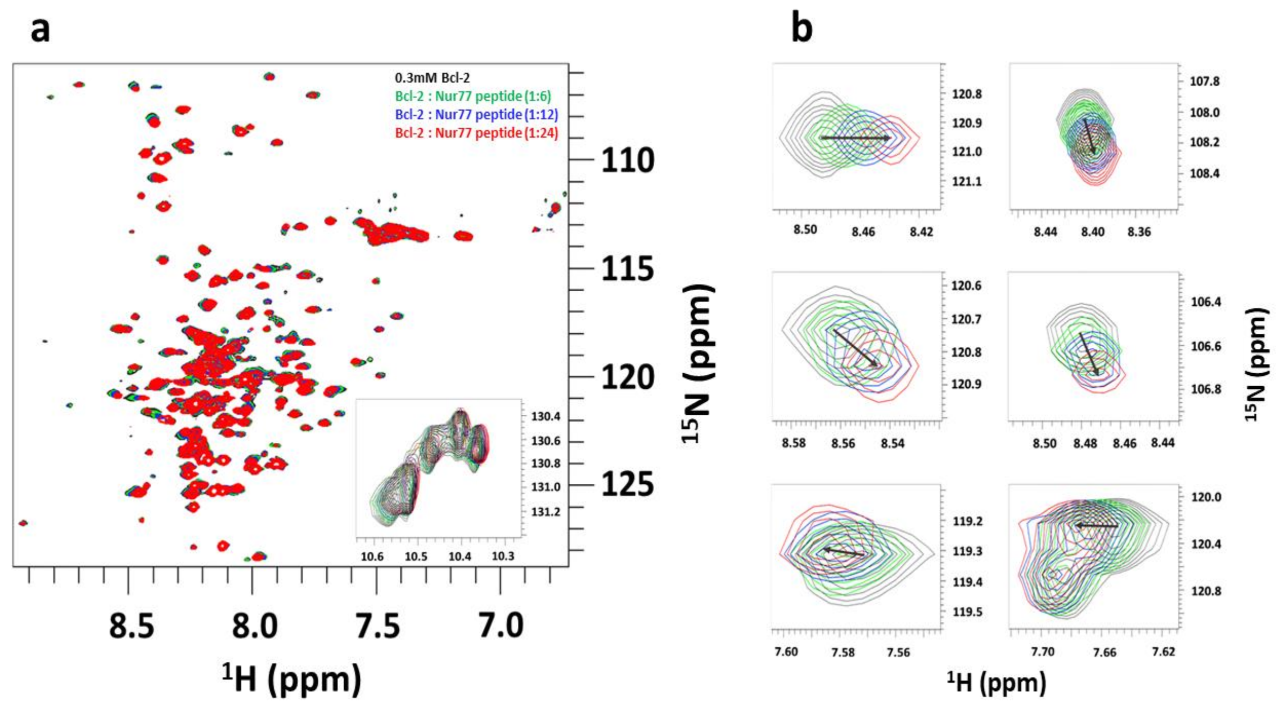

Supplementary Figure 4. Titration of Nur77 peptide against Bcl-2 protein. a Overlay of ^{1}H-^{15}N-TROSY-HSQC spectra showing chemical shift perturbations (CSP) observed in ^{15}N-labeled Bcl-2 upon titration with the Nur77 peptide. The spectrum of 0.3 mM Bcl-2 in 5 mM DPC micelles, in 20 mM NaPi, 20 mM NaCl, 2 mM TCEP at pH 6.0 is shown in black. Spectra of Bcl-2 with the Nur77 peptide added at 1:6, 1:12 and 1:24 molar ratio are shown in green, blue and red, respectively. **b** Zoomed boxes show the significantly perturbed peaks of the Bcl-2 spectra upon addition of the Nur77 peptide. K_D values range between 10 to 20 mM. Spectra were acquired at 310 K and at a ^{1}H frequency of 850 MHz.

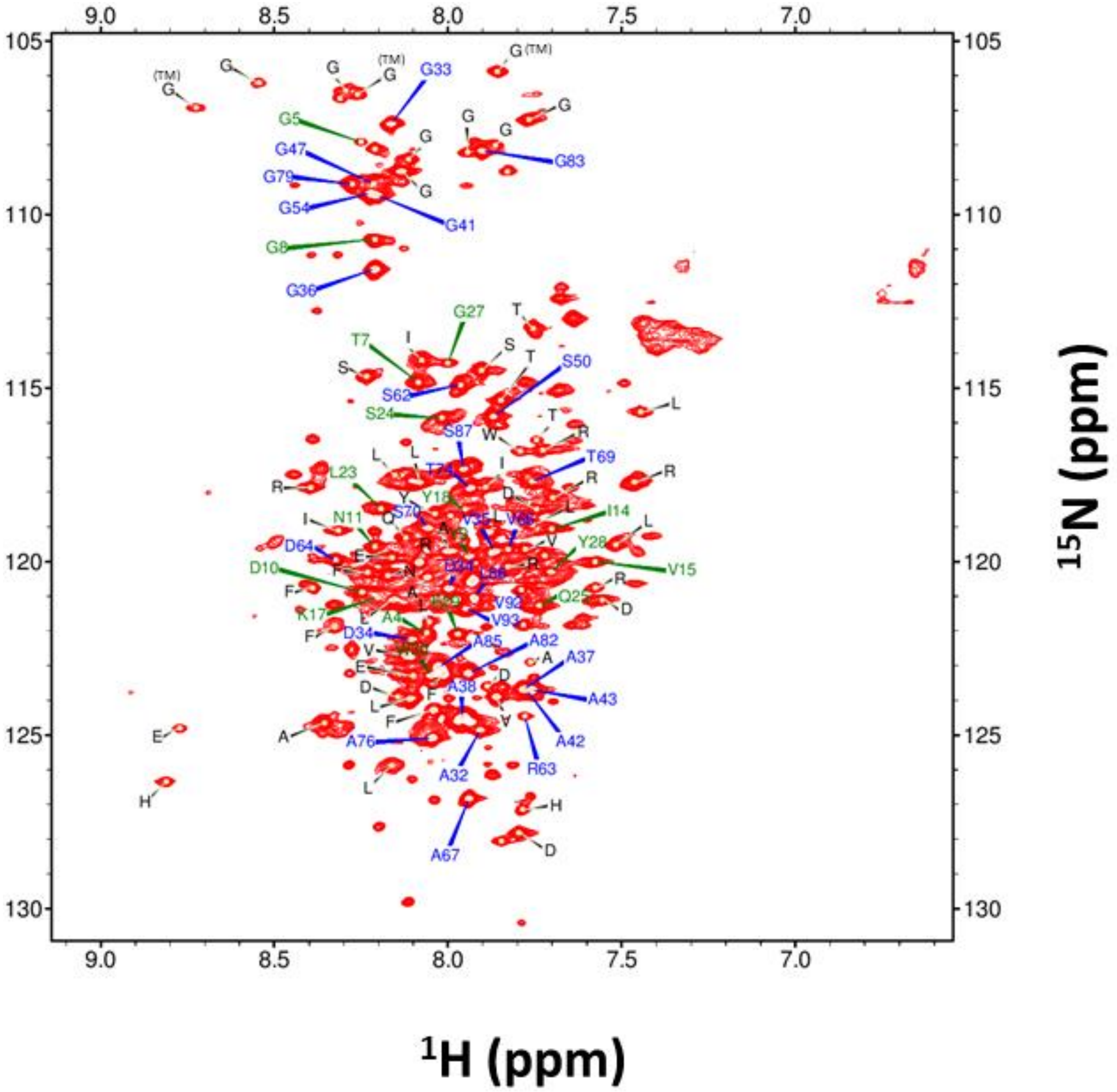

Supplementary Figure 5. Partial assignment of Bcl-2 residues. Resonance assignments of ^{13}C- and ^{15}N- labeled full-length Bcl-2 in DPC micelles (with assignments of residues of the flexible loop domain and the N-terminal shown in blue and green respectively) obtained from INEPT (insensitive nucleus enhancement polarization transfer) BEST-TROSY (Band-selective Excitation Short-Transient-Transverse Relaxation Optimized Spectroscopy) type triple-resonance experiments: HNCACB, HN(CO)CACB, HNCA, HN(CO)CA, HNCO and HN(CA)CO acquired at 328 K. Experimental details and data analysis are described in the main methods section.

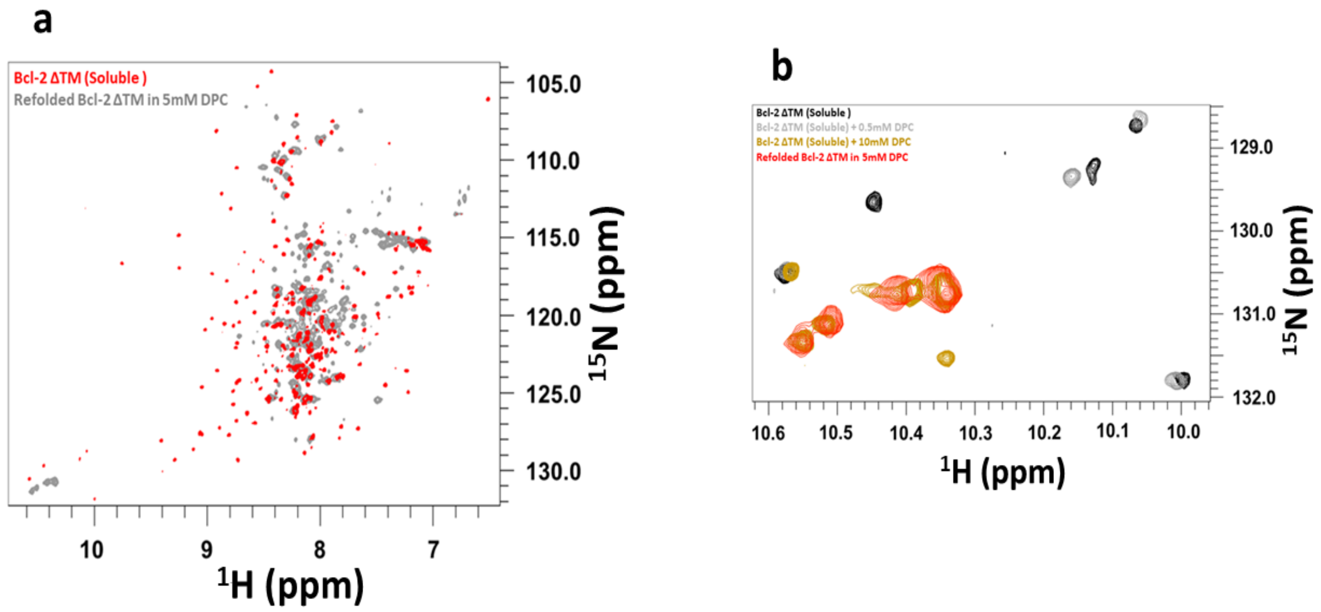

Supplementary Figure 6. ^{1}H-^{15}N TROSY-HSQC spectra of Bcl-2 ΔTM protein (without the transmembrane C-terminal helix). a Overlay of ^{1}H-^{15}N-TROSY-HSQC NMR spectra of the 0.7 mM ^{15}N-labeled Bcl-2 ΔTM in 5mM DPC micelles (in grey) in buffer (20 mM NaPi, 20 mM NaCl, 2 mM TCEP at pH 6.0) and 40 µM 15N-labeled Bcl-2 ΔTM in the same buffer (in red), respectively. **b** ^{1}H-^{15}N TROSY-HSQC spectra showing side-chain tryptophan $^{15}N\varepsilon^{1}H$ of 40 µM ^{15}N-labeled Bcl-2 ΔTM in buffer (black), 40 µM ^{15}N-labeled Bcl-2 ΔTM in 0.5 mM DPC micelles in buffer (grey), 40 µM ^{15}N-labeled Bcl-2 ΔTM in 10 mM DPC micelles in buffer (brown), and 0.7 mM refolded. ^{15}N-labeled Bcl-2 ΔTM in 5 mM DPC micelles in buffer. All spectra were measured at 298 K using a 850 MHz ^{1}H frequency.

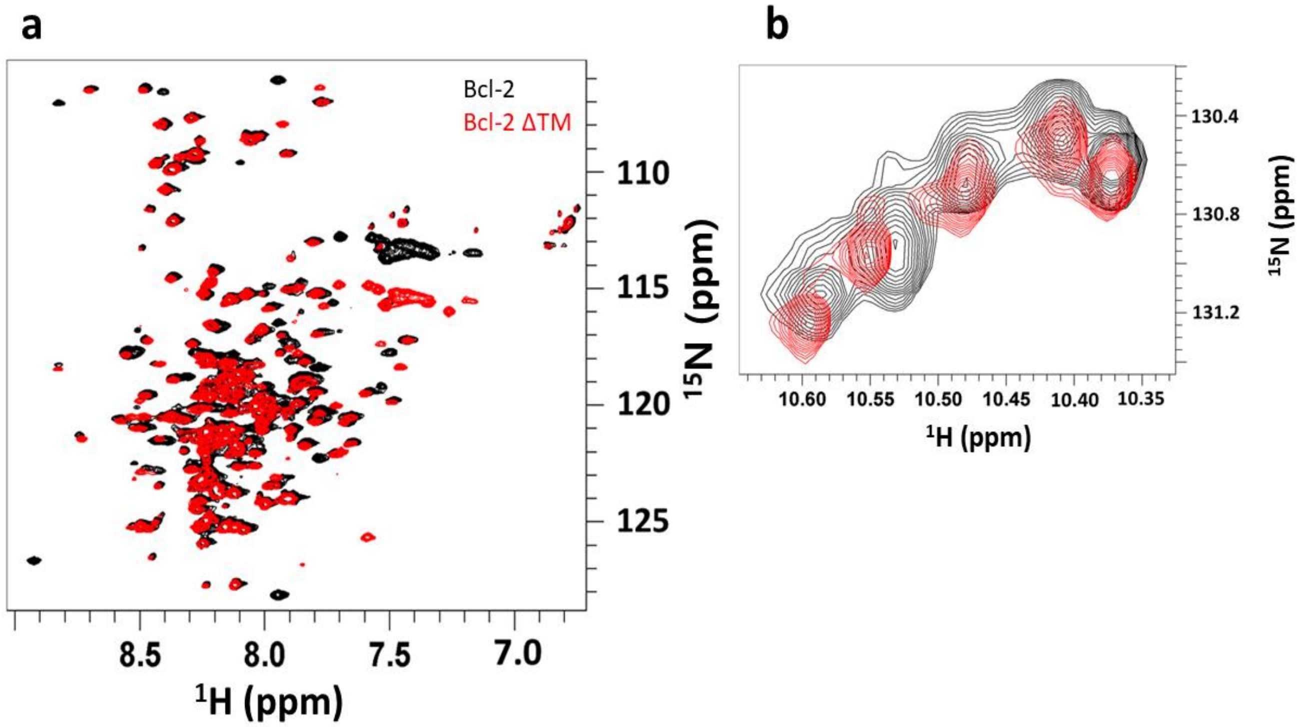

Supplementary Figure 7. Comparison of ^{1}H-^{15}N TROSY-HSQC spectra and tryptophan NMR signals of Bcl-2 and Bcl-2 ΔTM proteins in DPC micelles. a ^{1}H-^{15}N TROSY-HSQC spectra showing backbone ^{13}C-^{15}N-labeled 300 μM Bcl-2 in 5 mM DPC micelles in buffer (black) and 500 μM ^{15}N-labeled Bcl-2 ΔTM under same conditions (red). **b** Zoomed ^{15}Nε^{1}H region of side-chains of tryptophan of Bcl-2 and Bcl-2 ΔTM, respectively. All 2D ^{1}H-^{15}N TROSY experiments were performed with 16 or 32 number of scans, time-domain sizes of 256 (^{15}N) × 2048 (^{1}H) complex points and sweep widths of 11029.412 Hz and 2412.313 Hz or 2583.979 Hz along the ^{1}H and ^{15}N dimensions, respectively. All spectra were acquired using a ^{1}H frequency of 850 MHz.

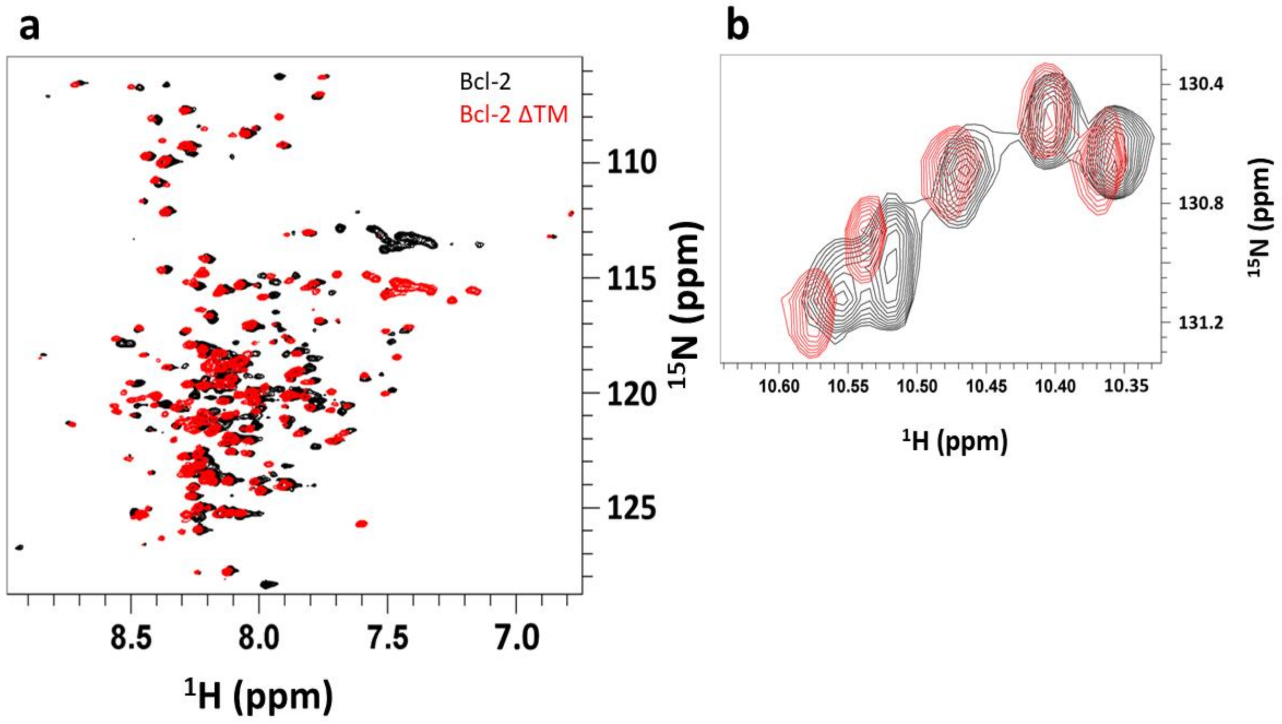

Supplementary Figure 8. Comparison between ^{1}H-^{15}N TROSY-HSQC spectra and tryptophan NMR signals of Bcl-2 and Bcl-2 ΔTM proteins in the presence of DPC below CMC (1.5 mM). a ^{1}H-^{15}N TROSY-HSQC spectra showing backbone of 35 μM ^{13}C-^{15}N-labeled Bcl-2 in 0.25 mM DPC micelles in buffer (black) and in 25 μM ^{15}N-labeled Bcl-2 ΔTM under the same conditions (red). **b** Zoomed ^{15}Nε^{1}H region of side-chains of tryptophan of Bcl-2 and Bcl-2 ΔTM, respectively. All 2D ^{1}H-^{15}N TROSY experiments were performed with 16 or 32 number of scans, time-domain sizes of 256 (^{15}N) × 2048 (^{1}H) complex points and sweep widths of 11029.412 Hz and 2412.313 Hz or 2583.979 Hz along the ^{1}H and ^{15}N dimensions, respectively. All spectra were acquired using a ^{1}H frequency of 850 MHz at 310 K.

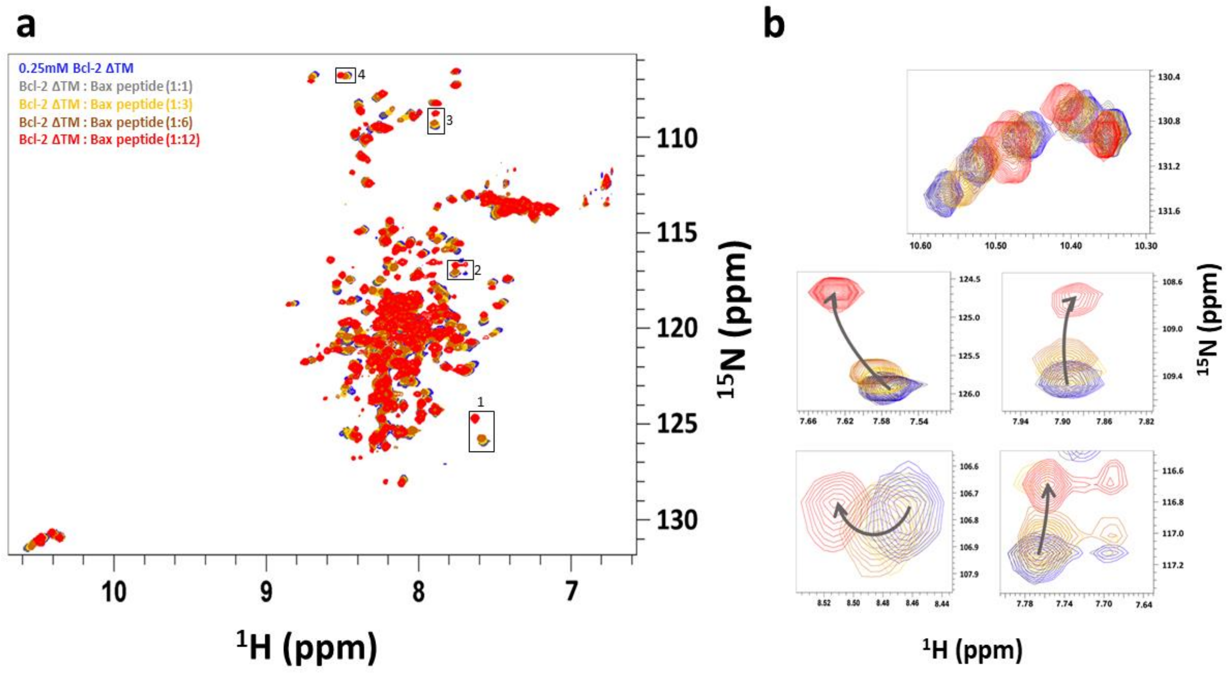

Supplementary Figure 9. **Titration of Bax-BH3 peptide against the Bcl-2 ΔTM variant.** **a** ^{1}H-^{15}N-TROSY-HSQC spectra showing observed chemical shift perturbations (CSP) of ^{15}N-labeled Bcl-2 ΔTM upon titration with the Bax-BH3 peptide. The spectrum of 0.25 mM Bcl-2 ΔTM in 5 mM DPC micelles in buffer is shown in blue. Spectra of Bcl-2 ΔTM with Bax-BH3 peptide added at 1:1, 1:3, 1:6 and 1:12 stoichiometry are shown in grey, yellow, brown and red, respectively. **b** Zoomed boxes show the significantly perturbed peaks of the full Bcl-2 ΔTM spectrum upon addition of the Bax-BH3 peptide. All spectra were acquired at 310 K and at a ^{1}H frequency of 600 MHz.

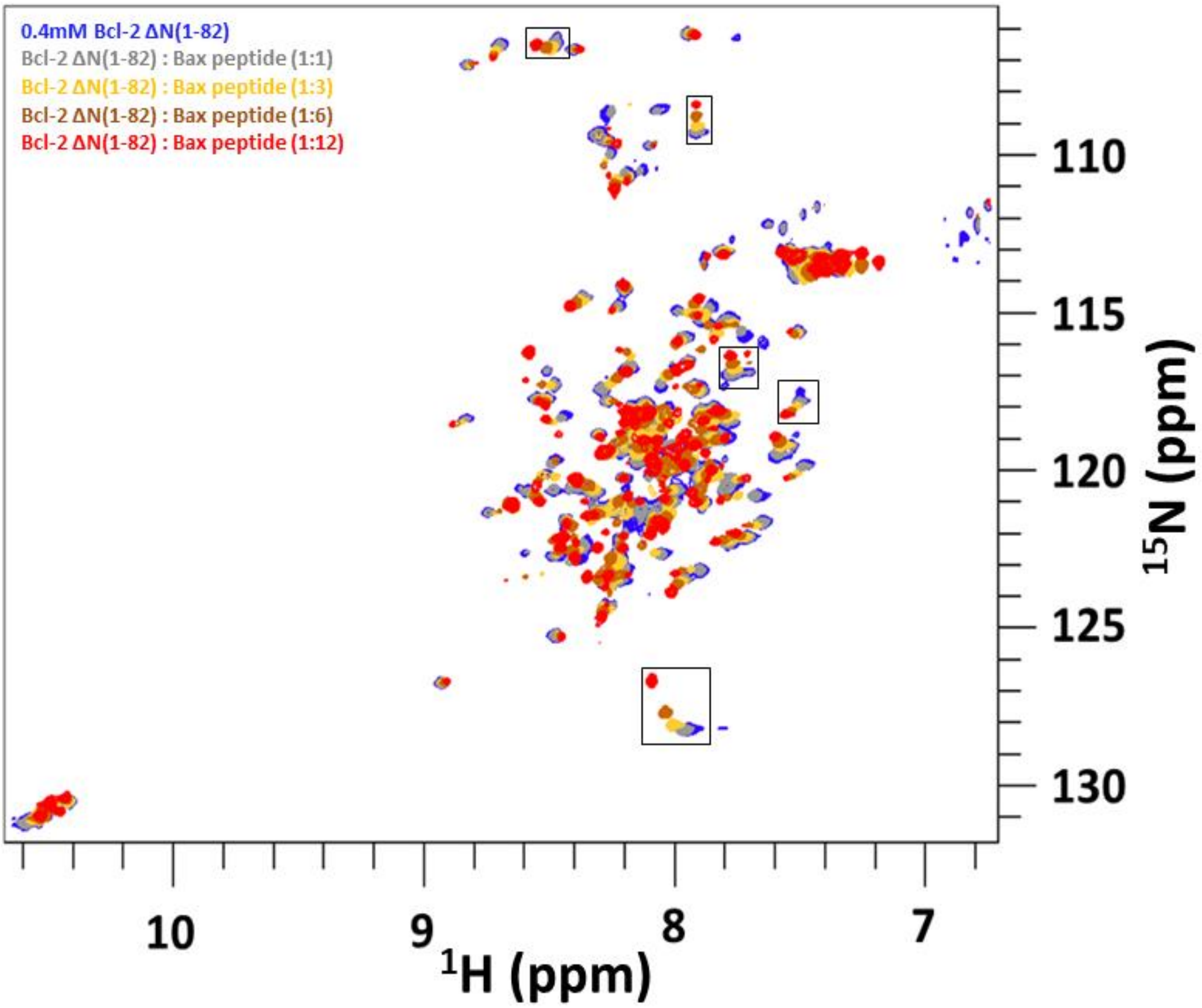

Supplementary Figure 10. **Titration of Bax-BH3 peptide against the Bcl-2 ΔN(1-82) variant.** ^{1}H-^{15}N-TROSY-HSQC spectra showing observed chemical shift perturbations (CSP) of ^{15}N-labeled Bcl-2 ΔN(1-82) upon titration with the Bax-BH3 peptide. The spectrum of 0.40 mM Bcl-2 ΔN(1-82) in 5 mM DPC micelles in buffer is shown in blue. Spectra of Bcl-2 ΔN(1-82)with Bax-BH3 peptide added at 1:1, 1:3, 1:6 and 1:12 molar ratio are shown in grey, yellow-orange, brown and red, respectively. Highlighted boxes show the significantly perturbed peaks observed in Bcl-2 and truncated variants upon addition of the Bax-BH3 peptide. All spectra were acquired at a ^{1}H frequency of 850 MHz at 310 K.

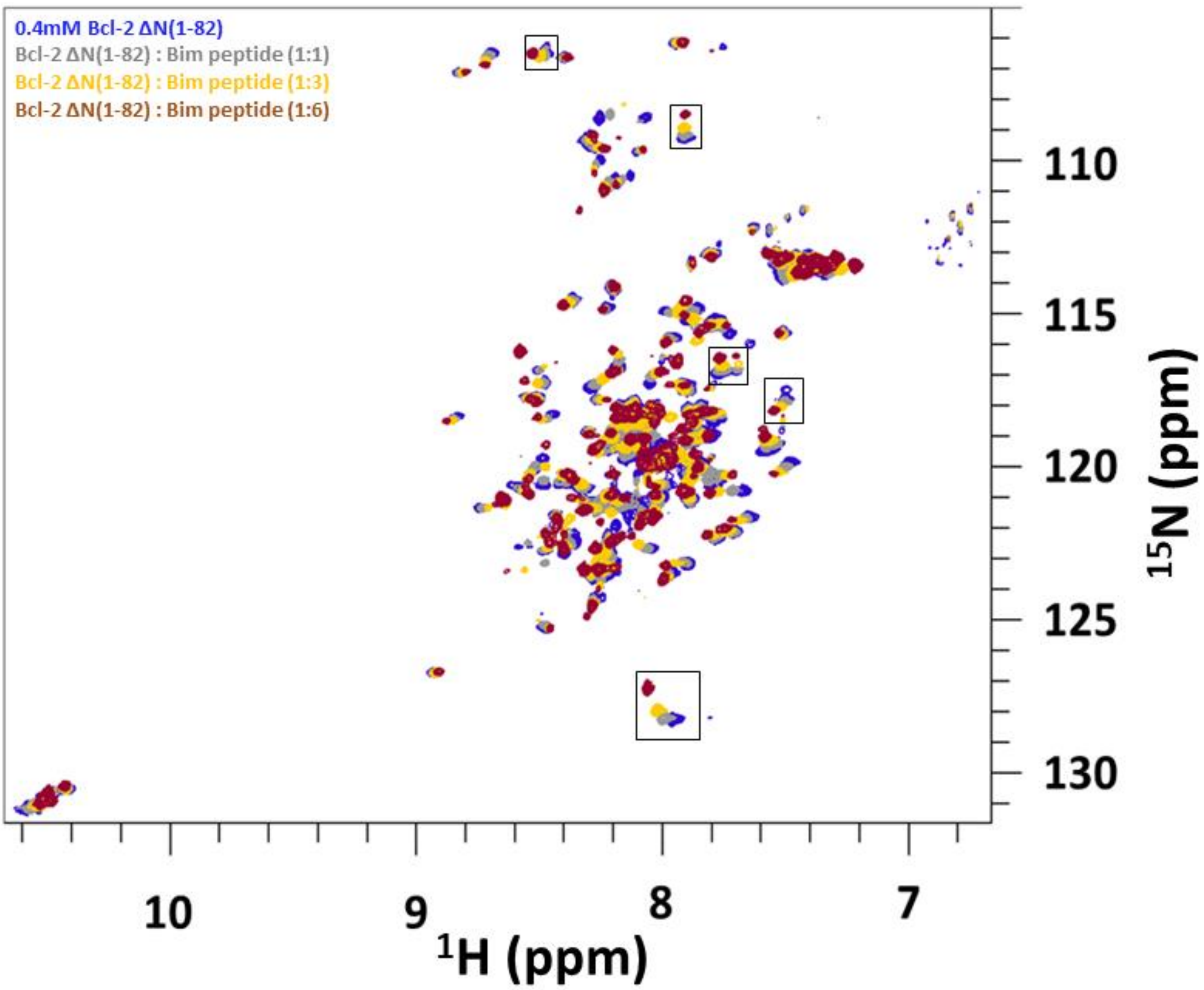

Supplementary Figure 11. **Titration of Bim-BH3 peptide against the Bcl-2 ΔN(1-82) variant.** ^{1}H-^{15}N-TROSY-HSQC spectra showing observed chemical shift perturbations (CSP) of ^{15}N-labeled Bcl-2 ΔN(1-82) upon titration with the Bim-BH3 peptide. The spectrum of 0.40 mM Bcl-2 ΔN(1-82) in 5 mM DPC micelles in buffer is shown in blue. Spectra of Bcl-2 ΔN(1-82) with Bim-BH3 peptide added at 1:1, 1:3 and 1:6 molar ratio are shown in grey, yellow-orange and brown, respectively. All spectra were acquired at a proton frequency of 850 MHz at 310 K.

Supplementary Table 1: Known and calculated Scattering Length Density (SLD) values for components of the interfacial samples used in the NR studies.

Sample component	SLD (ρ) / 10^{-6} Å^{-2}
h-DMPC tails[1]	-0.28
DMPC head group	1.98
d-Bcl-2 in H_2O	5.9
d-Bcl-2 in D_2O	7.5
h-Bcl-2 in D_2O	3.2
h-Bcl-2 in H_2O	1.9
Silicon oxide[1]	3.41
Silicon[1]	2.07
D_2O[1]	6.35
H_2O[1]	-0.56

[1]Known SLD values taken from Stidder *et al* [1], Clifton *et al* [2] and Foglia *et al* [3]. Calculated SLD values for proteins were acquired using the protein SLD calculator (http://psldc.isis.rl.ac.uk/Psldc/) and deuteration values obtained for the proteins in NMR studies.

Supplementary References

1. Stidder, B., Fragneto G. & Roser, S. T. Effect of low amounts of cholesterol on the swelling behavior of floating bilayers. *Langmuir* **21**, 9187-9193 (2005).
2. Clifton, L. A. et al. An accurate *in vitro* model of the E. coli envelope. *Angew. Chem. Int. Ed.* **54**,11952-11955 (2015).
3. Foglia, F. et al. Interaction of Amphotericin B with Lipid Monolayers. *Langmuir* **30**, 9147-9156 (2014).
