# Supplementary material for: Neutron reflectometry and NMR spectroscopy of full-length Bcl-2 protein reveal its membrane localization and conformation
Source: Commun Biol. 2021 Apr 27;4:507. doi: 10.1038/s42003-021-02032-1 (PMC8079415; doi:10.1038/s42003-021-02032-1)
Supplement: Supplementary file 2 — Reporting Summary [file 42003_2021_2032_MOESM2_ESM.pdf]

nature research

Corresponding author(s): Gerhard Gröbner

Last updated by author(s): Mar 6, 2021

Reporting Summary

Nature Research wishes to improve the reproducibility of the work that we publish. This form provides structure for consistency and transparency in reporting. For further information on Nature Research policies, see our Editorial Policies and the Editorial Policy Checklist.

Statistics

For all statistical analyses, confirm that the following items are present in the figure legend, table legend, main text, or Methods section.

n/a	Confirmed	
☒	☐	The exact sample size (*n*) for each experimental group/condition, given as a discrete number and unit of measurement
☒	☐	A statement on whether measurements were taken from distinct samples or whether the same sample was measured repeatedly
☒	☐	The statistical test(s) used AND whether they are one- or two-sided<br>*Only common tests should be described solely by name; describe more complex techniques in the Methods section.*
☐	☒	A description of all covariates tested
☐	☒	A description of any assumptions or corrections, such as tests of normality and adjustment for multiple comparisons
☐	☒	A full description of the statistical parameters including central tendency (e.g. means) or other basic estimates (e.g. regression coefficient) AND variation (e.g. standard deviation) or associated estimates of uncertainty (e.g. confidence intervals)
☒	☐	For null hypothesis testing, the test statistic (e.g. *F*, *t*, *r*) with confidence intervals, effect sizes, degrees of freedom and *P* value noted<br>*Give P values as exact values whenever suitable.*
☐	☒	For Bayesian analysis, information on the choice of priors and Markov chain Monte Carlo settings
☒	☐	For hierarchical and complex designs, identification of the appropriate level for tests and full reporting of outcomes
☒	☐	Estimates of effect sizes (e.g. Cohen's *d*, Pearson's *r*), indicating how they were calculated

Our web collection on statistics for biologists contains articles on many of the points above.

Software and code

Policy information about availability of computer code

Data collection	Mantidplot software was used to reduce experimental NR data sets.
Data analysis	Rascal NR software running under matlab was used to analyse NR data.

For manuscripts utilizing custom algorithms or software that are central to the research but not yet described in published literature, software must be made available to editors and reviewers. We strongly encourage code deposition in a community repository (e.g. GitHub). See the Nature Research guidelines for submitting code & software for further information.

Data

Policy information about availability of data

All manuscripts must include a data availability statement. This statement should provide the following information, where applicable:

- Accession codes, unique identifiers, or web links for publicly available datasets
- A list of figures that have associated raw data
- A description of any restrictions on data availability

The authors declare that all data supporting the findings of this study are available within the article, its supplementary information file and from the corresponding authors upon reasonable request. Neutron reflectometry data is directly available from 10.5286/ISIS.E.94115547 and 10.5286/ISIS.E.RB1720424 for experiment RB1810627 and RB1720424 respectively. Models used in NR analysis are available upon request.

Field-specific reporting

Please select the one below that is the best fit for your research. If you are not sure, read the appropriate sections before making your selection.

[x] Life sciences [ ] Behavioural & social sciences [ ] Ecological, evolutionary & environmental sciences

For a reference copy of the document with all sections, see nature.com/documents/nr-reporting-summary-flat.pdf

Life sciences study design

All studies must disclose on these points even when the disclosure is negative.

Sample size	Sample size was defined by the time available at the Neutron source and the experimental design to cover various parameters (like nature of lipid membranes, protein presence ...) and the available NMR time (where various replicates (different batches of Bcl-2) could be run.
Data exclusions	One NMR experiment was excluded due to protein degradation. It was finally replaced by a NMR experiment on a new batch of Bcl-2 protein which provided the same conclusion but without the presence of a minor fraction of degraded protein.
Replication	Due to the limited time on Neutron source (ISIS) we could only repeat the key sample measurements there (Bcl-2 in DMPC bilayers). Other membrane systems were tested but not replicated and not used in the study. Quality of Bcl-2 protein batches was controlled by NMR and also the structural integrity. Here, the different batches showed identical structural and functional behavior.
Randomization	Samples were run at neutron source after priority due to the limited time window. Similar strategy was used in NMR where samples were randomized in the way necessary if new batches of protein were needed.
Blinding	No Blinding

Reporting for specific materials, systems and methods

We require information from authors about some types of materials, experimental systems and methods used in many studies. Here, indicate whether each material, system or method listed is relevant to your study. If you are not sure if a list item applies to your research, read the appropriate section before selecting a response.

Materials & experimental systems

n/a	Involved in the study
[x]	[ ] Antibodies
[x]	[ ] Eukaryotic cell lines
[x]	[ ] Palaeontology and archaeology
[x]	[ ] Animals and other organisms
[x]	[ ] Human research participants
[x]	[ ] Clinical data
[x]	[ ] Dual use research of concern

Methods

n/a	Involved in the study
[x]	[ ] ChIP-seq
[x]	[ ] Flow cytometry
[x]	[ ] MRI-based neuroimaging
